# Characterization of Trap States in AlGaN/GaN MIS-High-Electron-Mobility Transistors under Semi-on-State Stress

**DOI:** 10.3390/nano14181529

**Published:** 2024-09-20

**Authors:** Ye Liang, Jiachen Duan, Ping Zhang, Kain Lu Low, Jie Zhang, Wen Liu

**Affiliations:** 1 School of Advanced Technology, Xi’an Jiaotong-Liverpool University, Suzhou 215123, China; 2 Department of Electrical Engineering and Electronics, University of Liverpool, Liverpool L69 3GJ, UK; 3 Department of Communications and Networking, Xi’an Jiaotong-Liverpool University, Suzhou 215123, China; 4 School of Chips, Entrepreneur College (Taicang), Xi’an Jiaotong-Liverpool University, Suzhou 215123, China

**Keywords:** AlGaN/GaN MIS-HEMT, current collapse, trap states, energy level, trap density

## Abstract

Devices under semi-on-state stress often suffer from more severe current collapse than when they are in the off-state, which causes an increase in dynamic on-resistance. Therefore, characterization of the trap states is necessary. In this study, temperature-dependent transient recovery current analysis determined a trap energy level of 0.08 eV under semi-on-state stress, implying that interface traps are responsible for current collapse. Multi-frequency capacitance–voltage (C-V) testing was performed on the MIS diode, calculating that interface trap density is in the range of $1.37\times 10^{13}$ to $6.07\times 10^{12}$ ${\mathrm{cm}}^{-2}$ ${\mathrm{eV}}^{-1}$ from $E_{C}-E_{T}=0.29$ eV to 0.45 eV.

## 1. Introduction

GaN-based devices are suitable for high-voltage and high-switching applications due to their wide bandgap and high carrier mobility of two-dimensional electron gas (2DEG) [1]. AlGaN/GaN high-electron-mobility transistors (HEMTs) suffer from current collapse, especially under high-drain bias in the off-state, due to surface defects related to N-vacancies [2].

There are some works dedicated to suppressing current collapse using physical methods, such as ECR $N_{2}$-plasma pre-treatment [2] and oxygen plasma treatment [3], or through structural optimization methods, such as the bi-passivation layer [4] and the fluorinated graphene passivation layer [5]. Some studies have found that Si_3_N_4_ can passivate the N-vacancies on the surface of AlGaN. However, it is not suitable as a gate-insulating layer due to its small band offset with AlGaN (bandgap E*_G_* of Si_3_N_4_ is approximately 5 eV; in comparison, the E*_G_* of AlGaN is approximately 4.1 eV) and relatively low dielectric constant ($\epsilon_{r}$∼7.5) [2]. Thus, many studies have proposed Al_2_O_3_ (E_G_∼7 eV and $\epsilon_{r}$∼9.3) as a gate dielectric and passivation layer [3,4,5,6,7,8,9,10,11]. These studies investigate the degradation mechanism under off-state stress in the device [2,3,4,5,11]; however, they rarely focus on the degradation under semi-on-state stress.

The semi-on-state stress condition is typically defined as a gate voltage higher than the threshold voltage but not exceeding two volts [12,13,14,15]. In this state, the drain voltage is always maintained at a high level, the average energy of the electrons is measured in terms of electron temperature (T*_e_*), the T*_e_* increases correspondingly, and the positions of hot spots also change [16]. Electron trapping could occur in the AlGaN barrier [17], the oxide layer, at the interface [13], or in the buffer layer [18]. This can lead to more severe current collapse or on-resistance degradation compared to off-state stress.

This study investigates the current collapse of AlGaN/GaN MIS-HEMTs with 20 nm Al_2_O_3_ as the gate dielectric and passivation under the semi-on and off-states by employing pulse I–V testing. Moreover, temperature-dependent transient recovery current tests and Arrhenius plots are performed to obtain the emission time constants and calculate the energy levels of interface traps associated with semi-on-state stress. Multi-frequency capacitance–voltage (C-V) testing was performed on the MIS diode to calculate interface trap density.

This article is organized into four sections. Section 2 describes the fabrication process of the device and its static characteristics. Section 3 presents the current collapse results of the device under semi-on and off-states, as tested by the pulse I–V method. Section 4 discusses the electron trapping mechanisms and calculates the energy levels and density of the interface traps. Conclusions are drawn in Section 5.

## 2. Device Fabrication and Characterization

**Figure 1:**
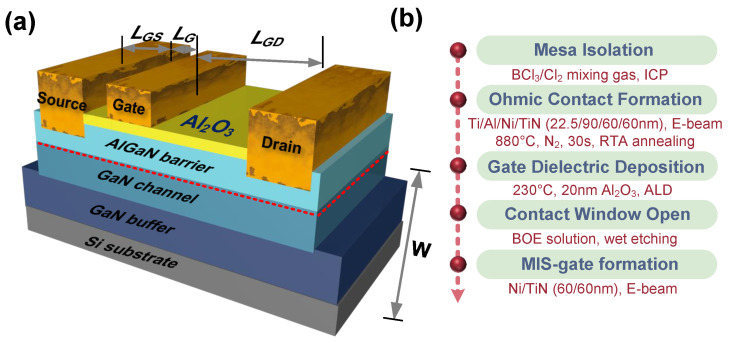
(**a**) The simplified schematic structure of AlGaN/GaN MIS-HEMTs with a 20 nm ALD-Al_2_O_3_ as gate dielectric and passivation. (**b**) The fabrication process of the device.

The simplified schematic structure of the AlGaN/GaN MIS-HEMTs, which was analyzed in this research, is depicted in Figure 1a, while the fabrication process is shown in Figure 1b. The devices are fabricated on a commercially available epitaxial wafer supplied by Enkris Semiconductor, Inc., Suzhou, China, with a sheet carrier density of $1\times 10^{13}$ ${\mathrm{cm}}^{-2}$. The wafer consisted of several layers: a 23 nm undoped ${\mathrm{Al}}_{0.25}$${\mathrm{Ga}}_{0.75}$N barrier layer, a 330 nm GaN channel layer, and a 5 μm undoped GaN buffer layer, all grown on the Si substrate.

**Figure 2:**
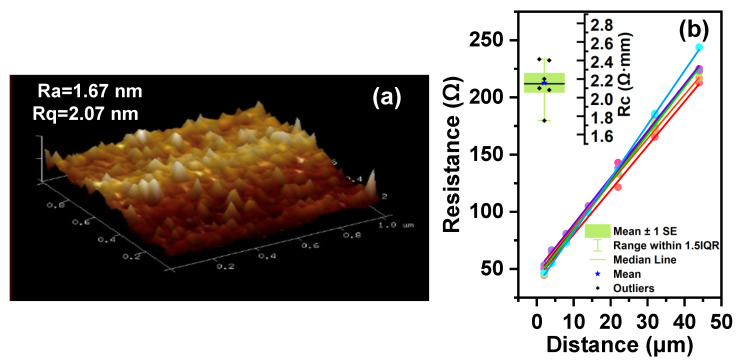
(**a**) Surface roughness after ICP etching. (**b**) Contact resistance was obtained using the TLM method.

The fabrication process starts with the mesa isolation step, achieved by an inductively coupled plasma (ICP) dry-etching system. The etching rate is 16 nm/min. The etching gas flow rates are ${\mathrm{Cl}}_{2}$/${\mathrm{BCl}}_{3}$ = 4/10 sccm, with the ICP power set at 50 W and the radio frequency (RF) power set at 30 W. A 350 nm PECVD-Si_3_N_4_ layer is used as a hard mask to protect the access region. After the etching process, the etched height is approximately 350 nm, with an average surface roughness (R*_a_*) of 1.67 nm and a root mean square roughness (R*_q_*) of 2.07 nm, as measured by atomic force microscopy (AFM), as shown in Figure 2a. To reduce leakage current and minimize native oxide and nitrogen vacancies at the GaN surface, the samples are immersed in an 80 °C tetramethylammonium hydroxide (TMAH) solution for 5 min.

Next, a metal stack of Ti/Al/Ni/TiN (22.5/90/60/60 nm) is evaporated using an electron-beam (E-beam) evaporation system. Then, the metal stack is annealed at 880 °C in an N_2_ atmosphere for 30 s to form the N-type ohmic contact. The contact resistances (R*_c_*) are measured using the transmission line model (TLM) by assessing the resistance between pairs of contacts with different spacings of 2, 4, 8, 14, 22, 32, and 44 μm [19]. As shown in Figure 2b, the average contact resistance is 2.1 Ω·mm, with a sheet resistance of 383 Ω/sqr.

Before the Al_2_O_3_ deposition, the samples were immersed in a 10% HCl solution for 1 min. After that, a 20 nm Al_2_O_3_ is deposited using the atomic layer deposition (ALD) system as the gate dielectric and passivation layer. In the ALD system, tetramethylaluminum (TMA) provides the aluminum source, while H_2_O provides the oxygen source, with pulse time of 50 ms and 40 ms, respectively. The deposition temperature is 230 °C, and the chamber pressure is 12 Pa. The deposition rate is 0.08 nm/cycle. The buffer oxide etch (BOE) solution is used to wet-etch the Al_2_O_3_ layer above the source and drain contact regions.

Finally, a Ni/TiN (60/60 nm) metal stack is evaporated as the gate electrode. The device dimensions are as follows: the distance between the source and drain ($L_{SD}$) is 28 μm; the distance between the gate and source ($L_{GS}$) is 5 μm; the gate length ($L_{G}$) is 3 μm; the distance between the gate and drain ($L_{GD}$) is 20 μm; and the device width (W) is 100 μm.

**Figure 3:**
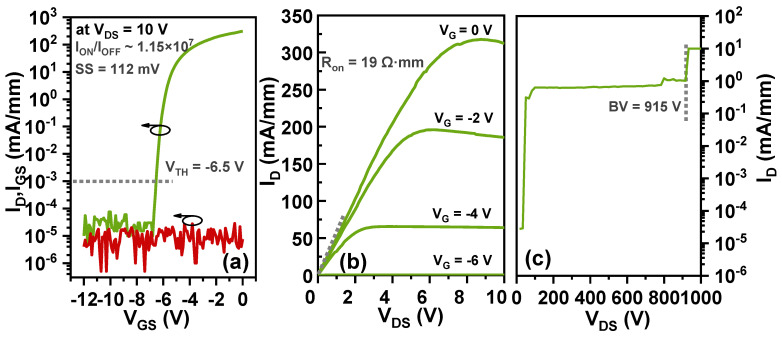
(**a**) Transfer characteristics ($I_{D}$-$V_{GS}$) and (**b**) output characteristics ($I_{D}$-$V_{DS}$) of the devices. (**c**) Off-state breakdown test results with a floating substrate.

Figure 3a,b illustrate the device’s static transfer and output characteristics by Agilent B1505A Medium Power Source Monitor Unit (MPSMU) and High-Power-Source Monitor Unit (HPSMU). In Figure 3a, the gate voltage sweeps from −12 V to 0 V with a step of 0.5 V, the tested device exhibits a satisfactory $I_{ON}$/$I_{OFF}$ ratio of $1.15\times 10^{7}$, a low gate leakage current level of $10^{-4}$ to $10^{-6}$ mA/mm, a subthreshold voltage swing (SS) of 112 mV, and a threshold voltage ($V_{TH}$) of −6.5 V at a drain current criterion of 1 μA/mm. In Figure 3b, the gate voltage increases from −12 V to 0 V in steps of 2 V, while the drain voltage sweeps from 0 V to 10 V in steps of 0.5 V. The drain current is 310 mA/mm at a gate voltage ($V_{GS}$) of 0 V, and a drain voltage ($V_{DS}$) of 10 V. The static on-resistance ($R_{ON,S}$) is 19 Ω·mm at a gate voltage ($V_{GS}$) of 0 V, and a drain voltage ($V_{DS}$) of 0.1 V.

In Figure 3c, the MPSMU provides the gate voltage, and the High-Voltage-Source Monitor Unit (HVSMU) provides the drain voltage. The gate voltage remains at −8 V (off-state), while the drain voltage increases from 0 V to 1000 V in steps of 5 V. The drain voltage at which a sudden increase in current occurs is called the breakdown voltage. The breakdown voltage (BV) of the device is 915 V at $V_{GS}$ of −8 V with a floating substrate.

## 3. Current Collapse Results

Current collapse phenomena are tested by the pulse I-V method; two Agilent B1500A High-Resolution-Source Monitor Units (HRSMUs) provide gate and drain pulses; the pulse width ratio of the stress phase to the sampling phase is $T_{stress}$/$T_{on}$ = 130 ms/500 μs. During the stress phase, the gate and drain voltage are defined as $V_{GS,0}$ and $V_{DS,0}$, respectively. The devices are subjected to two stress conditions: off-state stress (with $V_{GS,0}$= −8 V and $V_{DS,0}$ = 40 V) and semi-on-state stress (with $V_{GS,0}$ = −6 V and $V_{DS,0}$ = 40 V).

After the stress phase, the transient drain current $I_{D}$ is monitored at an on-state gate bias ($V_{GS,M}$) of 0 V and a drain-source voltage ($V_{DS,M}$) ranging from 1 V to 10 V. The on-resistance before the stress phase ($R_{ON,0}$) and after the stress phase (dynamic on-resistance $R_{ON,D}$) is determined by dividing $V_{DS,M}$ by the transient on-state current observed at $V_{GS,M}$ = 0 V and $V_{DS,M}$ = 1 V. The $R_{ON,D}$-to-$R_{ON,0}$ ratio represents the on-resistance degradation.

**Figure 4:**
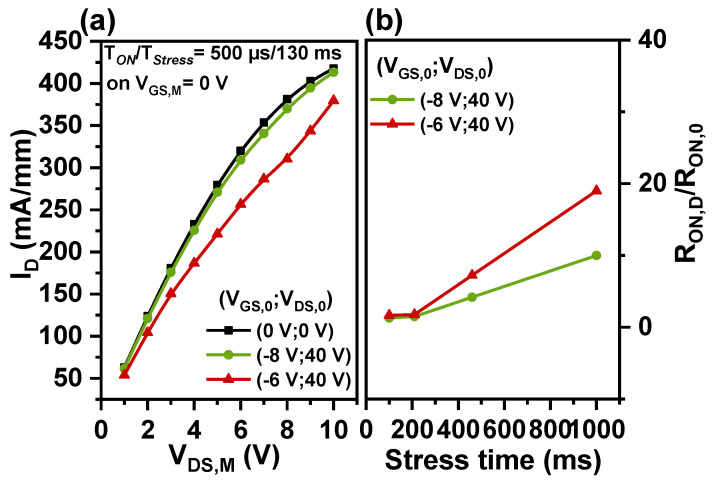
(**a**) Current collapse results are shown for off-state stress (green dotted line), semi-on-state stress (red dotted line), and no stress (black dotted line). (**b**) Changes in the R$R_{ON,D}$/$R_{ON,0}$ ratio with increasing stress time.

In Figure 4a, it is evident that the current collapse is more severe under semi-on-state stress than under off-state stress. The maximum $I_{D}$ decreases significantly, with a reduction of about 10% under semi-on-state stress and approximately 2% under off-state stress. In Figure 4b, the stress time increases from 100 ms to 1000 ms, while the sampling phase remains at 500 μs. It has been observed that with increased stress time, the transient current $I_{D}$ decreases, indicating an increase in dynamic on-resistance [20,21]. Before 200 ms of stress, on-resistance degradation is similar for both conditions. After 200 ms, dynamic on-resistance increases more under semi-on-state stress than under off-state stress. At 1000 ms, the $R_{ON,D}$-to-$R_{ON,0}$ ratio is 19 for semi-on-state stress and 10 for off-state stress.

Silvaco TCAD was used to model the device and simulate its electric field. To determine the high-density 2DEG channel, spontaneous and piezoelectric polarization models, the Shockley–Read–Hall (SRH) model [22], and Fermi–Dirac statistics were employed. The Albrecht model and the GaN velocity saturation model were used to characterize carrier behavior in low and high electric fields, respectively. Hot electron injection was modeled to assess the current under semi-on-state stress. The metal work function for the source and drain is 3.93 eV, while the work function for the gate is 5.05 eV. The GaN buffer is carbon-doped with a concentration of $5\times 10^{16}\;{\mathrm{cm}}^{-3}$.

**Figure 5:**
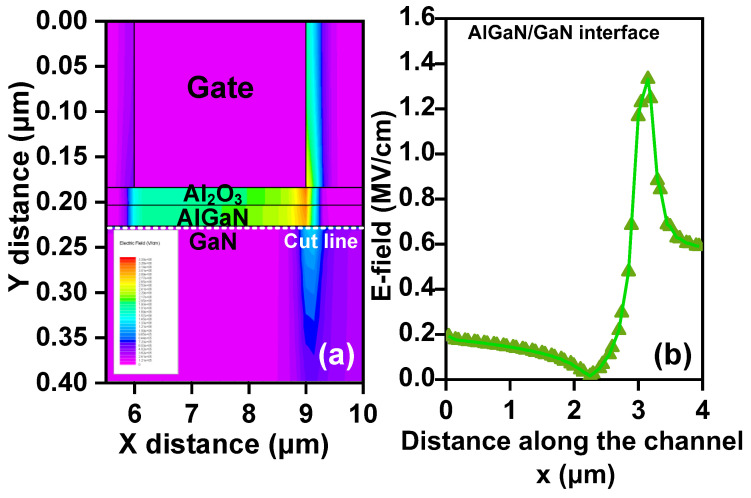
(**a**) Electrical field profile of AlGaN/GaN MIS-HEMT under semi-on-state stress. (**b**) Electrical field distribution along the 2DEG channel.

The electrical field profile of the device under semi-on-state stress ($V_{GS}$ = −6 V and $V_{DS}$ = 40 V) is shown in Figure 5a, and the distribution along the AlGaN/GaN interface (or at the 2DEG channel) is shown in Figure 5b. The electric field is concentrated in the oxide layer and AlGaN-layer region beneath the gate, with the highest electric field occurring at the edge of the gate on the drain side. Along the cut line at the 2DEG channel, the maximum electric field peak is about 1.3MV/cm.

## 4. Discussion

**Figure 6:**
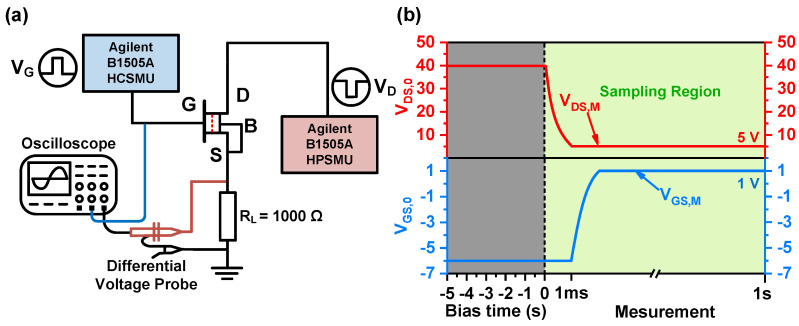
(**a**) Schematic setup for transient measurement. (**b**) The semi-on-state stress at ($V_{GS,0}$, $V_{DS,0}$) = (−6 V, 40 V) is applied to the samples for 5 s. Then, after the stress period, the transient current is obtained by measuring the voltage drop across the resistive load during the on-state ($V_{GS,M}$, $V_{DS,M}$) = (1 V, 5 V) for 1 s.

The temperature-dependent transient recovery current test investigates the location and distribution of trap levels responsible for $R_{ON}$ degradation under semi-on-state stress [23]. The test setup is shown in Figure 6a,b, the Agilent B1505A High-Current-Source Monitor Unit (HCSMU) and HPSMU provide the gate and drain pulses. Initially, a semi-on-state stress condition of ($V_{GS,0}$, $V_{DS,0}$) = (−6 V, 40 V) is applied to the device for 5 s to induce electron trapping. The transient recovery current is then monitored for 1 s under an on-state condition of ($V_{GS,M}$, $V_{DS,M}$) = (1 V, 5 V). A 1000 Ω resistor is connected to the device’s source electrode for this test. The test temperature is gradually raised from 25 °C to 150 °C, with a step of 25 °C. The temperature-dependent transient recovery current $I_{D}$ is calculated by detecting the real-time voltage difference across the resistor during device-switching.

**Figure 7:**
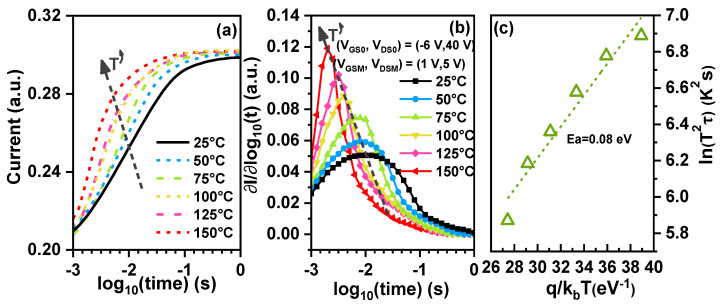
(**a**) The temperature-dependent transient recovery current results after semi-on-state stress. (**b**) Emission time constant spectra extracted from the temperature-dependent transient recovery current results. (**c**) Arrhenius plots calculate the activation energy of AlGaN/GaN MIS-HEMTs with Al_2_O_3_ as dielectric under semi-on-state stress.

The results are shown in Figure 7a. It has been found that at the starting point of 1 ms, the current density is not sensitive to temperature and remains at around 0.21. The saturation of the recovery current occurs earlier as the temperature increases, indicating that the device recovers faster at a higher temperature [24]. Figure 7b shows the extracted emission time constant (τ*_e_*) spectra for the transient recovery currents of the device. The τ*_e_* decreases with the temperature increase, from 11 ms to 1.9 ms, as the temperature rises from 25 °C to 150 °C. Figure 7c shows that the activation energy of traps is 0.08 eV below the conduction band. The fact that this activation energy is less than 0.1 eV indicates that the interface traps are the primary cause of $R_{ON}$ degradation [18,25].

Multi-frequency capacitance–voltage tests are performed on the MIS diode to determine the density of interface traps. The dielectric thickness is the same as that of MIS-HEMTs. The gate voltage is swept from −12 V to 3 V with a step of 50 mV. The AC small signal is 0.2 V, and the measurement frequency ($f_{m}$) is varied from 1 kHz to 1 MHz. As the gate voltage increases, two slopes reflect different interface characteristics. At $V_{GS}$ = −11 V, electrons accumulate in the 2DEG channel. At this point, the frequency dispersion originates from the AlGaN/GaN interface. Subsequently, at $V_{GS}$ from −11 V to −9 V, the capacitance increases until it reaches a constant value. This constant value is equal to the series capacitance of the dielectric and the AlGaN barrier layer. When the gate voltage is increased to 0 V, the capacitance increases again as electrons are transferred to the dielectric/semiconductor interface. At this point, the interface trap states exhibit frequency-dependent characteristics.

**Figure 8:**
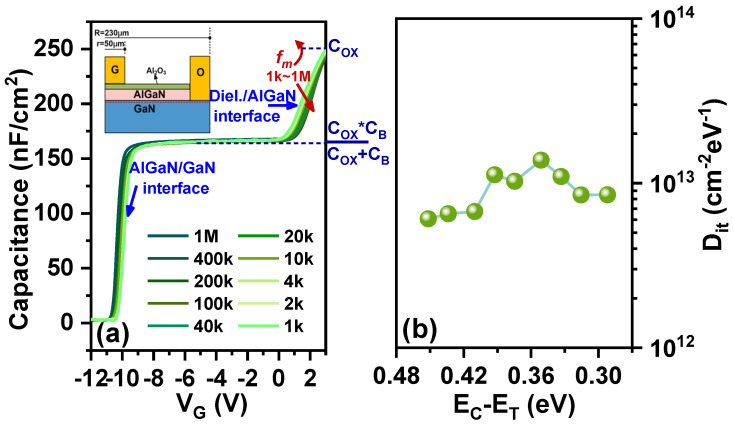
(**a**) Multi-frequency C-V characteristics of AlGaN/GaN MIS diode. (**b**) $D_{it}$-$E_{T}$ mapping in the MIS diode. Measurement frequency $f_{m}$ varies from 1 kHz to 1 MHz.

Figure 8a shows that the voltage difference at the start of the second slope ($V_{ON}$) corresponds to the interface traps responding at different frequencies [26,27]. $V_{ON}$ shifts positively with increasing frequency. The voltage dispersion ($\Delta V_{ON}$) observed at two measurement frequencies (f_1_ and f_2_) is attributed to the presence of interface traps within the energy range from $E_{trap(f1)}$ to $E_{trap(f2)}$. The energy level of the detectable interface trap, $E_{trap(fm)}$, as a function of the measurement frequency $f_{m}$, can be expressed as

(1) $$E_{trap(fm)}=E_{C}-E_{T}=kTln\frac{\nu_{th}\sigma_{n}N_{C}}{2\pi f_{m}}.$$

Here, *k* represents Boltzmann’s constant, *T* is the measurement temperature, $N_{C}=2.7\times 10^{18}$ ${\mathrm{cm}}^{-3}$ is the effective density of states in the conduction band of GaN, and $\sigma_{n}$ is the electron capture cross-section, assumed to be $1\times 10^{-14}\;{\mathrm{cm}}^{2}$ [28,29,30,31,32]. The thermal velocity of electrons, $\nu_{\mathrm{th}}$, is $2\times 10^{7}\;\mathrm{cm}\cdot s^{-1}$.

The interface trap density at different frequencies, as shown in Figure 8b, ranges from $1.37\times 10^{13}$ to $6.07\times 10^{12}\;{\mathrm{cm}}^{-2}\;{\mathrm{eV}}^{-1}$ for $E_{C}-E_{T}=0.29\;\mathrm{eV}$ to 0.45eV, indicating that interface traps closer to the conduction band edge have a higher density. This observation is consistent with findings reported in other studies [28,33,34,35,36,37,38].

The following Table 1 compares the MIS diode with different insulators and surface treatments. Specifically, it is important to note that this paper investigates the degradation of the device under semi-on-state stress, attributed to hot-electron and self-heating effects. Therefore, it is necessary to consider the variation in substrate materials, as different substrates have different thermal conductivity. For example, sapphire ($K_{Sapp}$ = 0.35 W/cm−K), Si ($K_{Si}$ = 1.5 W/cm−K), SiC ($K_{SiC}$ = 4.9 W/cm−K), and diamond ($K_{Dia}$ = 20 W/cm−K) [39]. Therefore, the following table compares the interface traps for different insulator materials under the same substrate (Si).

According to the above test results, the current collapse phenomena under semi-on-state stress are more severe than under off-state stress due to hot electrons being injected and trapped in the bulk or at the interface, as shown in Figure 4a [40]. Furthermore, we found that $R_{ON}$ degradation is more pronounced in devices with larger access areas due to more interface traps [41].

## 5. Conclusions

This work investigates the current collapse in AlGaN/GaN MIS-HEMTs with 20 nm Al_2_O_3_ as their gate dielectric under off-state and semi-on-state stress. Traps cause on-resistance degradation under semi-on-state stress in the bulk and at the interface. The energy level and density of interface traps are determined using temperature-dependent transient recovery current tests and multi-frequency C-V tests, with an energy level of 0.08 eV and an interface trap density ranging from $1.37\times 10^{13}\;\mathrm{to}\;6.07\times 10^{12}\;{\mathrm{cm}}^{-2}\;{\mathrm{eV}}^{-1}$ for $E_{C}-E_{T}=0.29\;\mathrm{eV}\;\mathrm{to}\;0.45\;\mathrm{eV}$.

## Figures and Tables

**Table 1:** Comparison of interface trap density and energy level of different insulators and surface treatments on Si substrate.

Insulator	Surface Treatment	Test Method	Interface Density (eV^−1^cm^−2^)	Energy Level (eV)
Al_2_O_3_ [28]	O_2_ plasma	Multi-frequency capacitance–voltage	9.1 × $10^{12}$~4.8 × $10^{12}$	0.28 to 0.47
Al_2_O_3_ [28]	Octadecanethiol	Multi-frequency capacitance–voltage	6.1 × $10^{12}$~3 × $10^{12}$	0.28 to 0.47
Al_2_O_3_ [33]	N_2_ plasma	Multi-frequency capacitance–voltage	6 × $10^{12}$~6 × $10^{11}$	0.24 to 0.78
Al_2_O_3_ (This work)	HCl solution	Multi-frequency capacitance–voltage	1.37 × $10^{13}$~6.07 × $10^{12}$	0.29 to 0.45
ZrO_2_ [34]	HCl solution	Multi-frequency capacitance–voltage	4.7 × $10^{13}$~9.4 × $10^{12}$	0.28 to 0.47
AlN [35]	In situ low-damage plasma	C-V hysteresis	2.0 × $10^{13}$	No mention
SiN_x_ [36]	HF: H_2_O solution	Gated Hall method	2.3 × $10^{13}$~4 × $10^{12}$	1.2 to 2.3
S_3_N_4_ [37]	HCl solution	High-frequency capacitance–voltage	1.4 × $10^{12}$~2.8 × $10^{11}$	0.53 to 0.71
LaHfOx [38]	Rapid thermal annealing at the gate recess region	C-V hysteresis	7.5 × $10^{11}$	No mention

## Data Availability

Data are contained within the article.
